# Post-concussion Vulnerability to Transient Global Amnesia

**DOI:** 10.3389/fneur.2020.517863

**Published:** 2020-11-12

**Authors:** Matthew D. Garvey, Caitlin J. Miller, Esther U. Kim, George Skulikidis, Teena Shetty

**Affiliations:** Department of Neurology, Hospital for Special Surgery, New York, NY, United States

**Keywords:** transient global amnesia (TGA), amnesia, concussion, mTBI (mild traumatic brain injury), cerebral vulnerability, cerebral blood flow (CBF)

## Abstract

Few studies have investigated transient global amnesia (TGA) in the context of a concussion and the concussion sequelae following TGA. Here we review the case of a 43-year-old male with onset of transient global anterograde and retrograde amnesia 22 days after a sustained concussion. The patient's head CT, MRI of brain, and EEG were reported normal, and the patient regained full cognitive function 8 h after the TGA episode, with no recollection of the conspiring events. Following the TGA episode, the patient experienced notable worsening of concussive symptoms, including headache, head pressure, anxiety, neck pain, feeling slowed down, fogginess, not feeling right, difficulty remembering, and fatigue. The patient remained symptomatic for 32 days after the TGA episode. We suggest that a lingering window of post-concussion cerebral vulnerability, which may extend beyond clinical recovery, could lead to increased susceptibility to acute cognitive deficits, such as TGA.

## Introduction

### Background

Transient global amnesia (TGA) is a rare clinical syndrome, which affects predominantly patients aged 50–70 years, characterized by an acute onset of anterograde and retrograde amnesia, often accompanied by repetitive questioning, lasting up to 24 h, and not associated with other neurological deficits. The annual incidence of TGA is 3.4–10.4 per 100,000 people (1). Proposed mechanisms include vascular (e.g., venous flow disturbances or focal arterial ischemia), epileptic, hypoxic, or migraine-related pathogenesis (1). Although clinical characteristics of TGA have been studied for over 50 years, true etiology remains unknown. Furthermore, few studies have investigated TGA in the context of concussion and the clinical sequelae following TGA. We present a case report of a 43-year-old male with TGA following mild traumatic brain injury (mTBI), with evidence supporting the role of mTBI as a precipitating event facilitating the pathophysiological cascade of TGA.

### Case Presentation

A 43-year-old left-handed male working in finance presented to the emergency room (ER) with transient global anterograde and retrograde amnesia 22 days after sustaining a concussion. In regard to the concussive incident, the patient sustained trauma to the front left side of his head while skiing and experienced no loss of consciousness or amnesia. The patient sought out the ski patrol and medical personnel at the ski lodge. He immediately reported feeling “shaken up” and “foggy,” and experienced disrupted sleep the night of the injury. He returned to a normal work routine 2 days post-injury and over the next week reported improvement in fogginess but heightened anxiety. One week post-injury, he resumed “strenuous exercise” without exacerbation of symptoms. The patient had no prior personal or family history of head injury, headache, migraine, anxiety, or depression.

On the day of the TGA episode, the patient was asymptomatic except for a lingering concussion symptom of heightened anxiety. The post-concussion anxiety is notable given reports in literature that cite the association of psychological and physiological stress preceding TGA (2, 3). The TGA episode occurred immediately after a cold shower, which was preceded by a swim in a cold pool, physical activity with use of an inversion board, and television viewing, respectively. On the day of the TGA episode, he presented to the ER with tremors, anxiety, and anterograde and retrograde amnesia characterized by repetitive questioning. On exam, he was alert, oriented to person, and able to recognize his wife, but could not name the president, year, or identify his surroundings. Head CT and MRI of the brain (whole-brain T1-weighted, T2-weighted, diffusion-weighted, and susceptibility-weighted sequences) were performed ~3 h after admission, with neuroimaging and EEG all reported as normal.

The patient regained full cognitive function after 8 h, with no recollection of the episode. Post-TGA, he experienced intermittent headaches of 2–3/10 severity, described as a band of pressure around the forehead, which lasted for 2 weeks. The lingering post-concussion anxiety was still present and unchanged in severity. Other symptoms reported to have developed in the days after TGA were all mild in severity and included neck pain, feeling slowed down, fogginess, not feeling right, difficulty remembering (described as a vague sense of slower retrieval of memories rather than the short-term memory deficit seen in TGA), and fatigue. Of note, these symptoms were self-reported on a concussion symptom evaluation checklist and not representative of impaired cognition. The patient was seen for multiple neurological follow-up visits where memory, naming, repetition, comprehension, and ability to follow command tasks were administered by a board-certified neurologist (TS). At each visit, he was able to complete the tasks accurately, demonstrating fully intact mental status. He was deemed fully recovered from his concussion with no persistent symptoms 32 days after the TGA episode (54 days after initial head injury).

## Discussion

While its exact pathogenesis remains unknown, TGA has been associated with both physical and emotional precipitating events, such as physical exertion, changes in body temperature (i.e., cold water immersion), and anxiety—each present in the current case (2, 3). While these events have commonly been implicated as risk factors for TGA (1–3), there have only been a handful of TGA episodes precipitated by mild traumatic brain injury reported in the literature, all predating the 2000s and occurring in younger patients (4–7). Although previous literature excludes patients with recent head trauma, as per the Hodges and Warlow (8) diagnostic criteria for TGA, there is not a strong consensus for the temporal definition of “recent,” which is critical as the patient presented here was diagnosed with a concussion 22 days prior to the TGA episode. Moreover, the definition of mTBI has evolved over recent years, but the symptoms are rather distinct from those of TGA, and the chronology and course in relation to the head injury distinguishes the two as different disease entities. In our case study, differential diagnosis clearly excluded head trauma as the direct source of the transient global amnesia.

The TGA episode began immediately following cold water immersion, a frequently cited triggering event for TGA (3). This occurred in the context of other remote vulnerability factors to TGA, such as persistent anxiety related to the sustained concussion. Here we attempt to elucidate the relationship between concussion and increased vulnerability to TGA, by focusing on the mTBI variables—such as heightened stress response and cerebral blood flow (CBF) dysfunction—which may precipitate and ultimately facilitate the cascade of TGA. It is also important to note that clinical presentation of mTBI is retrospective and biographical in nature, which may exclude other potentially salient risk factors.

Precipitating events to TGA may be divided into both remote factors, which can precede TGA by several days to weeks, and immediate factors occurring minutes prior to the TGA event (3). In a study of 113 TGA patients, a fifth of patients experienced a stress-related event and heightened anxiety prior to the episode (2). Moreover, a study by Quinette et al. further revealed the association between anxiety and TGA through analysis of longer-term remote anxiety in patients (such as conflict at home or work, health problems, or money worries), which was often noted prior to TGA in the study patient population (3). Preceding the TGA episode, the patient presented here reported heightened anxiety, a remote psychological stress event to TGA and a commonly endorsed symptom of concussion (9).

The deleterious effects of physiological stress (i.e., cortisol) on the hippocampus are thought to lead to changes in synaptic plasticity, structural alterations, and functional impairments in CA1 neurons (10). Additionally, multiple neuroimaging studies have shown transient white matter lesions in the CA1 subfield in a significant amount of TGA patients 24–115 h post-symptom onset (2, 11, 12). However, timing of neuroimaging in our case study did not allow for detection of such lesions, as MRI was performed during the amnestic phase to aid in the differential diagnosis of TGA. From a mechanistic point of view, a stress response may lead to increased vulnerability to TGA through dysfunction of CA1 cells in the hippocampus. This further supports the association of anxiety, TGA, and cerebral vulnerability after concussion.

One of the notable consequences of mTBI is the dysregulation of CBF in the acute, post-acute, and chronic stages after injury, and beyond clinical recovery (13). Interestingly, venous flow disturbance is also a leading hypothesis for the pathogenesis of TGA (1). While CBF was not assessed in our case study, it is possible that altered CBF after concussion may also increase vulnerability to TGA in susceptible individuals. Additional neuroimaging studies including CBF before, during, and after the TGA episode and serial MRI for transient white matter lesions in the hippocampal CA1 region would be helpful to further understand TGA in the context of concussion and increased cerebral vulnerability.

Lingering cerebral vulnerability should be considered as a potential pathophysiological mechanism in instances of TGA following head injury, as patients may still be in a critical window of recovery. It is not clear to what extent concussion sequelae, not limited to heightened anxiety, may facilitate the pathophysiological cascade of TGA. Cerebral dysfunction following concussion and transient changes in cerebral blood flow, potentially associated with precipitating events such as anxiety (14), cold water immersion (15), and concussive symptoms, should be further investigated as they relate to increased brain susceptibility to acute cognitive deficits, such as TGA.

## Ethics Statement

This study was approved and carried out in accordance with the recommendation of Hospital for Special Surgery Institutional Review Board. Written informed consent was obtained from the individual(s) for the publication of any potentially identifiable images or data included in this article.

## Author Contributions

TS contributed to study concept and design, drafting of the manuscript, and case-study supervision. MG, CM, EK, and GS contributed to drafting the manuscript and intellectual content not limited to study design and case interpretation. All authors contributed to the article and approved the submitted version.

## Conflict of Interest

TS reports the following commercial or financial relationships: GE-NFL, Abbott, Perseus, Teva Pharmaceuticals, Chembio Diagnostics, Marker AG, and GE-NFL Medical Advisory Board. The remaining authors declare that the research was conducted in the absence of any commercial or financial relationships that could be construed as a potential conflict of interest.
